# Effectiveness of Behaviour Therapy for Children and Adolescents with Tourette Syndrome and Chronic Tic Disorder in a Naturalistic Setting

**DOI:** 10.1007/s10578-020-01098-y

**Published:** 2020-12-14

**Authors:** Per Andrén, Vera Wachtmeister, Julia Franzé, Caroline Speiner, Lorena Fernández de la Cruz, Erik Andersson, Elles de Schipper, Daniel Rautio, Maria Silverberg-Mörse, Eva Serlachius, David Mataix-Cols

**Affiliations:** 1 Department of Clinical Neuroscience, Karolinska Institutet, Child and Adolescent Psychiatry Research Center, Gävlegatan 22, 113 30 Stockholm, Sweden; 2 Stockholm Health Care Services, Region Stockholm, Stockholm, Sweden; 3 Department of Clinical Neuroscience, Division of Psychology, Karolinska Institutet, Stockholm, Sweden

**Keywords:** Tourette syndrome, Tic disorders, Behaviour therapy, Exposure with response prevention, Habit reversal training

## Abstract

It is unclear if the results of randomised controlled trials (RCTs) of behaviour therapy (BT) for Tourette syndrome (TS) and chronic tic disorder (CTD) can be generalised to naturalistic clinical settings and are durable long-term. In this naturalistic study, 74 young people with TS/CTD received BT at a specialist clinic. Data were collected at baseline, post-treatment, and at 3-, 6-, and 12-month follow-ups. Measures included the Yale Global Tic Severity Scale (YGTSS) and the Clinical Global Impression-Improvement scale (CGI-I), amongst others. Tic severity and tic-related impairment improved after treatment, with large within-group effect sizes. At post-treatment, 57% of the participants were classified as treatment responders according to the CGI-I. Tic severity and tic-related impairment improved further through the follow-up, with 75% treatment responders at the 12-month follow-up. BT is an effective and durable treatment for young people with TS/CTD in a naturalistic specialist clinical setting, with comparable effects to RCTs.

**Electronic supplementary material:**

The online version of this article (10.1007/s10578-020-01098-y) contains supplementary material, which is available to authorized users.

## Introduction

Tourette syndrome (TS) and chronic (motor or vocal) tic disorder (CTD) are neurodevelopmental disorders characterised by the presence of motor and/or vocal tics lasting for at least 1 year [1]. TS/CTD are associated with functional impairment in multiple domains, including everyday life and activities, social adjustment, and academic performance [2, 3]. Additionally, TS/CTD are also associated with high rates of psychiatric comorbidity, which further add to the patients’ burden [4].

There are several treatment options available for patients with TS/CTD. Treatment guidelines from both Europe and North America recommend behaviour therapy (BT) as the first-line intervention, based on a combination of clinical trial evidence and expert consensus [5–7]. Out of several available BT modalities, *comprehensive behavioural intervention for tics* (CBIT) has most evidence to date [8]. CBIT, which includes *habit reversal training* (HRT) as its primary component, has been successfully evaluated in several randomised controlled trials (RCTs) in both paediatric and adult samples [9–11]. There is also some evidence for the use of *exposure with response prevention* (ERP) [12]. European and Canadian treatment guidelines recommend both CBIT/HRT and ERP as first-line interventions for patients with TS/CTD, whilst American treatment guidelines primarily recommend CBIT/HRT [5–7].

Efficacy trials aim to evaluate treatment effects under extensively controlled conditions, which entail several methodological advantages and generate high internal validity. However, the efforts for achieving high internal validity might result in substantial deviations from regular clinical practice, affecting the external validity and the generalisability of results. Examples of factors contributing to low external validity in efficacy trials include using specific participant eligibility criteria (e.g., excluding participants with certain psychiatric or somatic comorbidities), delivering an intervention in a highly standardised way (e.g., using specific treatment protocols with a pre-determined number of sessions and highly experienced/trained therapists), restricting concurrent interventions (e.g., excluding individuals on psychotropic medications) or using extensive resources to maximize patient compliance with the intervention (e.g., reminding patients about appointments) [13]. For example, the two largest RCTs of BT for TS/CTD excluded participants with comorbid autism and used specific treatment protocols and highly trained therapists, although allowed for the inclusion of participants with most other psychiatric comorbidities and on concurrent tic medication (if stable) [9, 11].

Effectiveness trials (sometimes referred to as *pragmatic trials*), on the other hand, aim to evaluate treatment effects when delivered in regular clinical practice. To ensure generalisability of the results to the full spectrum of the population to which the treatment might be applied, the treatments are delivered more flexibly, the patients’ compliance may vary, and there are generally no strict inclusion and exclusion criteria [14]. Perhaps unsurprisingly, meta-analyses including studies from a variety of fields indicate that treatment effects are generally larger in efficacy trials, compared to effectiveness trials [15, 16].

To our knowledge, only one study has evaluated the effectiveness of BT for young people with TS/CTD [17]. In a retrospective chart review conducted at a specialised paediatric clinic, CBIT was found to be potentially effective in reducing tic severity in a small sample of patients (N = 10). However, larger studies are needed to more thoroughly evaluate how the results from previous efficacy trials [9, 11, 12] generalise to naturalistic clinical settings.

Another common issue in many studies of BT for TS/CTD is that follow-ups have been relatively short (up to 6 months after the end of treatment) [9, 11, 12] or restricted to initial treatment responders only [9, 11]. The few available long-term follow-up studies have generally been small (N= 23–32) [10, 18]. Long-term follow-ups are especially important in this patient group given that tics are highly influenced by contextual factors and have a natural tendency to wax and wane over time [1].

The aim of this study was two-folded. First, we evaluated the effectiveness of BT in a consecutive sample of youth with TS/CTD referred to a specialist clinic. Second, we investigated whether the treatment gains were maintained at long-term follow-up (12 months after the end of the treatment).

## Methods

### Study Design and Setting

This was an open longitudinal study in a naturalistic clinical setting. The study was conducted at a single site, a specialist outpatient clinic for obsessive–compulsive and related disorders, including TS/CTD, within the child and adolescent mental health services (CAMHS) in Stockholm, Sweden. The Swedish health care system is mainly government-funded and universal for all citizens. Our specialist clinic primarily receives referrals from local CAMHS services (Stockholm region), but in some cases also from other parts of Sweden. All consecutive patients referred to the clinic with a diagnosis of TS or CTD (together with their parents/legal guardians, henceforth referred to as parents) were invited to participate in the study, and those agreeing provided written informed consent. The study was approved by the Swedish Ethical Review Authority (reference number: 2015/1977-31/4 [2019-02132]).

### Procedure

Before their initial assessment at the clinic, all patients and their parents completed multiple standardised self- and parent-reported questionnaires online (see section *Time Points and Outcome Measures*). The initial assessment consisted of a 3-h evaluation at the clinic, where experienced clinical psychologists conducted a full psychopathological screening using the Mini-International Neuropsychiatric Interview for Children and Adolescents (MINI-KID) [19] and supplementary modules for obsessive–compulsive and related disorders. Each patient was then discussed by a multidisciplinary team of clinical child psychologists, child psychiatrists, and nurses, using all collected information from the interviews and questionnaires to diagnose patients according to the 10th revision of the International Statistical Classification of Diseases and Related Health Problems (ICD-10) [20] and the 5th edition of the Diagnostic and Statistical Manual of Mental Disorders (DSM-5) [1]. After the initial assessment, patients were either offered treatment at our specialist clinic, referred to more suitable services (e.g., according to other clinical priorities) or discharged (e.g., if symptoms were too mild or in remission). Treatment started as soon as a therapist was available, usually within a few weeks.

### Time Points and Outcome Measures

Data were collected at intake for all families. For patients who received treatment at the specialist clinic, data were also collected directly after treatment (post-treatment) and at 3-, 6-, and 12-month follow-up post-treatment. Because this was a naturalistic study, some patients were still in follow-up when the final data extraction was performed on 30 April 2020, which accounted for some of the data missing at later time points.

Data collection included a range of clinician-, self-, and parent-reported measures (see below). All measures were repeatedly collected at all time points. Clinician-rated measures were administered by the clinicians responsible for the initial assessment (baseline) as well as by the therapists providing treatment (all subsequent time points). All self- and parent-reported questionnaires were administered digitally via an online service.

#### Clinician-Rated Measures

The Yale Global Tic Severity Scale (YGTSS) [21] is a clinician-rated semi-structured interview which assesses tic severity and tic-related impairment. It is the most commonly used outcome measure in TS/CTD clinical trials [22]. The interview consists of three major parts: a symptom checklist, a tic severity rating, and a tic-related impairment rating. The total score of 0–100 points can be divided into several subscores, namely the Motor Tic Severity Score (0–25 points), the Vocal Tic Severity Score (0–25 points), the Total Tic Severity Score (YGTSS-TTSS; the two former scores combined, 0–50 points), and the Impairment Score (0–50 points). The YGTSS has demonstrated good psychometric properties [21, 23, 24].

The Clinical Global Impression-Improvement scale (CGI-I) [25] is a single-item clinician rating of symptom improvement. In this study, the CGI-I provided a rating of tic disorder symptom improvement compared to the baseline time point, with a range from 1 (‘very much improved’) to 7 (‘very much worse’). In line with previous TS/CTD trials [9, 11], ratings of 1 (‘very much improved’) and 2 (‘much improved’) were used to define treatment response.

The Children’s Global Assessment Scale (CGAS) [26] was also administered to provide a clinician rating of general functioning (not only restricted to tic disorder symptoms). The CGAS consists of a single item ranging from 1 to 100, where 100 indicates the best possible functioning.

#### Self-Reported Measures

The Premonitory Urge for Tics Scale (PUTS) is a 9-item (9-36 points) measure of premonitory urges. The questionnaire has shown good psychometric properties [27, 28]. The Gilles de la Tourette Syndrome-Quality of Life scale (GTS-QOL) [29] is a 27-item (0–108 points) disease-specific measure of health-related quality of life. Since the child and adolescent versions of the questionnaire had not yet been published when the data collection started, the adult version was used in this study. The Obsessive Compulsive Inventory-Child Version (OCI-CV) [30] is a 21-item (0–42 points) multi-dimensional measure of obsessive–compulsive symptoms. The questionnaire has demonstrated good psychometric properties [30]. Both the Children’s Depression Inventory-Short version (CDI-S; 10 items, 0–20 points) [31] and the Short Mood and Feelings Questionnaire-Child Version (SMFQ-CV; 13 items, 0–26 points) [32] were used in the study as self-reported measures of depressive symptoms, with the SMFQ-CV gradually replacing the CDI-S over time. For analyses, both depression measures were combined using *z*-scores. Lastly, the Work and Social Adjustment Scale-Youth version (WSAS-Y) [33], a psychometrically sound, 5-item (0–40 points) measure of functional impairment in several areas, was included.

#### Parent-Reported Measures

The Parent Tic Questionnaire (PTQ) [34] is a parent-rated measure of tic severity consisting of two separate lists of common motor and vocal tics. For each present tic, parents rate its frequency and intensity, generating a total score ranging from 0 to 224 points. The questionnaire has established psychometric properties [34]. Parents also rated their children’s depressive symptoms with the SMFQ-Parent Version (SMFQ-PV; 13 items, 0–26 points) [32] and global functional impairment with the WSAS-Parent version (WSAS-P; 5 items, 0–40 points) [33].

### Interventions

BT was delivered according to published treatment manuals [35, 36] further supplemented by locally developed patient worksheets. The choice of BT modality (HRT or ERP) was primarily according to therapist preference, even though the patient and their family were also invited to express their preference. In HRT, the patients practiced to become more aware of their tics and to perform *competing responses* (i.e., voluntary behaviours physically incompatible with the tics) aimed at interrupting tic occurrence. In ERP, patients practiced to suppress tics (response prevention) for increased periods of time. In addition, patients intentionally provoked their *premonitory urges* (i.e., unpleasant sensations typically preceding tic occurrence) to make tic suppression more difficult (exposure), with the aim of increasing their tic suppression abilities. Along with psychoeducation and the core elements of HRT or ERP, additional therapeutic strategies could be added by the therapist, such as relaxation training and interventions based on functional assessment (as included in CBIT) [35]. BT treatments were by default 10 sessions long, but could be tailored (shortened or extended) to each patient’s needs. Each therapy session generally lasted 1 h. Therapists were clinical psychologists (5 years of master level studies and a minimum of 1 year of clinical training) trained in BT with extensive experience in the treatment of obsessive–compulsive and related disorders, including TS/CTD, or trainee psychologists or clinical psychology master students under supervision of a senior clinical psychologist. Throughout the study period, more senior clinical psychologists with extensive experience in delivering BT for TS/CTD were readily available for consultation.

Additionally, some patients were on medication for their tics. This included prescriptions prior to being referred to the clinic, as well as new prescriptions by a child and adolescent psychiatrist at the clinic, according to clinical judgement and treatment guidelines.

### Statistical Analyses

Mixed-effects regression models for repeated measures using maximum likelihood estimation (MLE) of parameters were implemented on all continuous outcome measures. The models included fixed effects of time and random intercepts for the participant effects. The primary model included the baseline and post-treatment time points. Furthermore, to investigate the durability of the treatment effects, a second model was fitted which included the post-treatment, 3-, 6-, and 12-month follow-up time points. For graphical representation purposes only, a third model was also fitted including all time points from baseline to the 12-month follow-up. Significance thresholds (two-tailed) were set to *p *< 0.05. Bootstrapped within-group effect-sizes (*d*), derived from mixed-effects regression models, were calculated with the m_effectsize command in Stata developed by the Karolinska Institutet Biostatistics Core and available at www.imm.ki.se/biostatistics/stata. All analyses were performed using Stata 14.2 (StataCorp LLC). Sample sizes may vary for some of the analyses as a result of missing data. Missing data points are specified for each measure in all tables.

## Results

### Study Participants and Treatment Completion

**Fig. 1:**
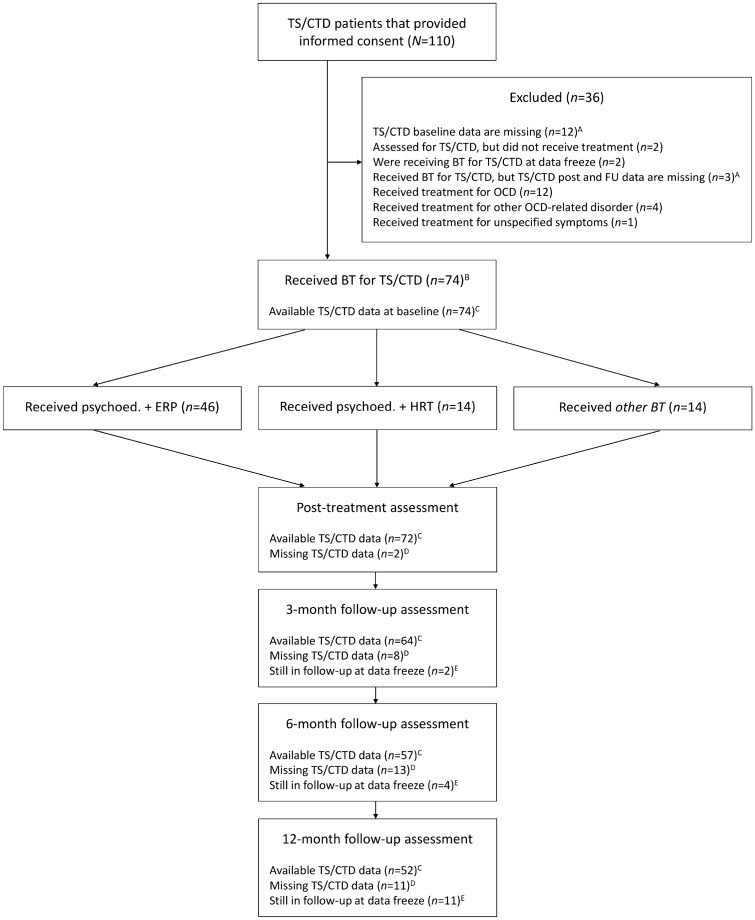
Study participants’ flow. *A* Participants were only excluded if *all* of the following data were missing at the specified time point: Yale Global Tic Severity Scale, Parent Tic Questionnaire, Premonitory Urge for Tics Scale, and Gilles de la Tourette Syndrome-Quality of Life scale; *B* During the study period, two participants received two (non-overlapping) rounds of treatment and 12-month long-term follow-up. In these cases, only the first round was included in the study; *C* Data are listed as available if *any* of the following data were available at the specified time point: Yale Global Tic Severity Scale, Parent Tic Questionnaire, Premonitory Urge for Tics Scale, and Gilles de la Tourette Syndrome-Quality of Life scale; *D* Data are listed as missing if *all* of the following data were missing at the specified time point: Yale Global Tic Severity Scale, Parent Tic Questionnaire, Premonitory Urge for Tics Scale, and Gilles de la Tourette Syndrome-Quality of Life scale; *E* Participants were still in follow-up at the time of the data freeze, hence they never reached this time point. *BT* behaviour therapy, *CTD* chronic tic disorder, *ERP* exposure with response prevention, *FU* 3-, 6-, and 12-month follow-up time points, *HRT* habit reversal training, *OCD* obsessive–compulsive disorder, *post* post-treatment time point, *psychoed* psychoeducation, *TS* Tourette syndrome

Figure 1 shows the study participants’ flow. A total of 110 patients with TS/CTD provided informed consent for the study between 1 January 2015 and 27 January 2020. Seventy-four participants received BT for TS/CTD at the specialist clinic, of which 46 received psychoeducation plus ERP, 14 received psychoeducation plus HRT, and 14 received *other BT*: psychoeducation plus a combination of ERP and HRT (n = 7) or psychoeducation plus ERP/HRT treatment rationales only (n = 7; when judged sufficient for the participant’s clinical needs). The 74 participants completed an average of 7.07 (SD = 3.01, range = 2–16) BT sessions between baseline and the post-treatment time point. Figure 1 further shows the availability of TS/CTD data for each time point. For the 74 participants, follow-up data continued to be collected until 30 April 2020.

Table 1 shows baseline demographic and clinical characteristics for the total BT sample and by BT modality. Supplementary Table S1 further shows the proportion of specific motor and vocal tics at baseline, according to the YGTSS symptom checklist.

**Table 1:** Baseline demographic and clinical characteristics of the total BT sample and by BT modality

	Total BT (*N *= 74)	ERP (*n *= 46)	HRT (*n *= 14)	Other BT (*n *= 14)
Age, mean (SD); min–max	11.36 (2.81); 6.42-17.83	11.11 (2.55); 6.42-16.83	12.14 (3.34); 7.42-17.50	11.41 (3.07); 6.42-17.83
Males, *n* (%)	52 (70)	32 (70)	9 (64)	11 (79)
Age of tic onset, mean (SD); min–max (*n *= 73)	5.79 (2.15); 2-14	5.62 (1.60); 3-9	6.50 (3.63); 2-14	5.64 (1.74); 3-8
Tic disorder, *n* (%)				
Tourette syndrome^A^	62 (84)	40 (87)	12 (86)	10 (71)
Chronic motor tic disorder	10 (14)	4 (9)	2 (14)	4 (29)
Chronic vocal tic disorder	2 (3)	2 (4)	0 (0)	0 (0)
Comorbidity, *n* (%)				
Any comorbidity	37 (50)	23 (50)	6 (43)	8 (57)
Attention-deficit/hyperactivity disorder	17 (23)	12 (26)	2 (14)	3 (21)
Obsessive-compulsive disorder	10 (14)	8 (17)	0 (0)	2 (14)
Anxiety disorders	8 (11)	3 (7)	3 (21)	2 (14)
Autism spectrum disorder	8 (11)	4 (9)	2 (14)	2 (14)
Depression	2 (3)	1 (2)	0 (0)	1 (7)
Dyslexia	2 (3)	2 (4)	0 (0)	0 (0)
Intellectual disability	1 (1)	0 (0)	1 (7)	0 (0)
Trichotillomania	1 (1)	1 (2)	0 (0)	0 (0)
Previous BT for TS/CTD, *n* (%)	9 (12)	4 (9)	1 (7)	4 (29)
Medication status, *n* (%)				
None	53 (72)	34 (74)	11 (79)	8 (57)
Stimulant	12 (16)	9 (20)	0 (0)	3 (21)
Melatonin	7 (9)	5 (11)	1 (7)	1 (7)
SSRI	7 (9)	4 (9)	1 (7)	2 (14)
Antipsychotic	4 (5)	1 (2)	1 (7)	2 (14)
Antihistamine	3 (4)	2 (4)	0 (0)	1 (7)
α2-agonist	1 (1)	1 (2)	0 (0)	0 (0)
Additional support in school, *n* (%) (*n *= 67)	25 (37)	18 (42)	3 (23)	4 (36)
Parent education^B^, *n* (%) (*n *= 54)				
College/University	48 (89)	31 (89)	9 (90)	8 (89)
Secondary school	5 (9)	3 (9)	1 (10)	1 (11)
Primary school	1 (2)	1 (3)	0 (0)	0 (0)
Family history of TS/CTD, 1st degree, *n* (%)	32 (43)	17 (37)	8 (57)	7 (50)
Family history of TS/CTD, 2nd degree, *n* (%)	17 (23)	12 (26)	1 (7)	4 (29)

*BT* behaviour therapy, *CTD* chronic motor or vocal tic disorder, *ERP* exposure with response prevention, *HRT* habit reversal training, *SD* standard deviation, *SSRI* selective serotonin reuptake inhibitor, *TS* Tourette syndrome

^A^ This includes two participants who were diagnosed with provisional tic disorder at baseline and eventually fulfilled Tourette syndrome criteria during the follow-up period

^B^ The highest level of education among pairs of parents was selected

### Clinician-Rated Measures and Treatment Response

Mixed-effects regression analyses at post-treatment showed a significant reduction on the YGTSS-TTSS for the total BT sample (coefficient [95% CI] − 7.74 [− 9.33 to − 6.16], *p *< 0.001), as well as for each BT modality (ERP: − 7.90 [− 9.81 to − 5.99], *p *< 0.001; HRT: − 6.09 [− 9.87 to − 2.31], *p *= 0.002; *other BT*: − 8.81 [− 12.72 to − 4.91], *p *< 0.001). Bootstrapped effect sizes (*d*) were the following: 1.03 (0.78 to 1.29) for the total sample, 1.09 (0.75 to 1.43) for the ERP group, 0.82 (0.19 to 1.44) for HRT, and 1.13 (0.59 to 1.66) for patients receiving *other BT*. Table 2 shows detailed statistics for the YGTSS and other TS/CTD-specific measures at post-treatment for the total BT sample. Supplementary Tables S2–S4 show the equivalent information separately for each BT modality.

**Table 2:** Detailed statistics for TS/CTD-specific measures at post-treatment for the total BT sample

	Total BT (*N *= 74)		
	Mean (SE)^A^	Within-group difference	Within-group effect size^B^
		Coefficient (95% CI); *p*-value	Bootstrapped *d* (95% CI)
Yale global tic severity scale			
Total tic severity score			
Baseline (*n *= 71)	23.43 (0.87)		
Post-treatment (*n *= 71)	15.68 (0.87)	− 7.74 (− 9.33 to − 6.16); *p *< 0.001*	1.03 (0.78 to 1.29)
Motor tic severity score			
Baseline (*n *= 71)	14.26 (0.48)		
Post-treatment (*n *= 71)	9.56 (0.48)	− 4.70 (− 5.69 to − 3.70); *p *< 0.001*	1.13 (0.78 to 1.48)
Vocal tic severity score			
Baseline (*n *= 71)	9.18 (0.59)		
Post-treatment (*n *= 71)	6.11 (0.59)	− 3.07 (− 4.07 to − 2.07); *p *< 0.001*	0.61 (0.37 to 0.84)
Impairment score			
Baseline (*n *= 71)	21.84 (1.07)		
Post-treatment (*n *= 71)	9.27 (1.07)	− 12.57 (− 14.70 to − 10.45); *p *< 0.001*	1.37 (1.04 to 1.70)
Parent tic questionnaire			
Baseline (*n *= 69)	37.61 (2.27)		
Post-treatment (*n *= 53)	22.97 (2.49)	− 14.64 (− 19.28 to − 10.01); *p *< 0.001*	0.76 (0.50 to 1.01)
GTS-quality of life scale			
Baseline (*n *= 57)	31.24 (2.24)		
Post-treatment (*n *= 40)	16.51 (2.59)	− 14.73 (− 20.01 to − 9.45); *p *< 0.001*	0.86 (0.57 to 1.14)
Premonitory urge for tics scale			
Baseline (*n *= 67)	19.95 (0.75)		
Post-treatment (*n *= 46)	20.02 (0.87)	0.74 (− 1.65 to 1.80); *p *= 0.933	− 0.01 (− 0.36 to 0.34)

*BT* behaviour therapy, *CI* confidence interval, *CTD* chronic motor or vocal tic disorder, *GTS* Gilles de la Tourette, *SE* standard error, *TS* Tourette syndrome

^A^ Estimated means from the mixed-effects regression model

^B^ Bootstrapped effect sizes (*d*) are derived from the mixed-effects regression model. Effect sizes of 0.2, 0.5, and 0.8 are considered small, moderate, and large, respectively

*Significant at an alpha level of 0.05

At post-treatment, 38 out of 74 participants (57%, available data for *n *= 67) were classified as treatment responders according to the CGI-I. Numbers were too small to meaningfully report on the specific BT modalities separately.

Between baseline and the post-treatment time point, 8 out of 74 participants (11%) changed their medication (α2-agonists or antipsychotics): 4 participants (5%) either increased their dosage or started medication, 2 participants (3%) switched from one compound to another, and 2 participants (3%) either decreased their dosage or stopped taking medication for their tics. A mixed-effects regression analysis excluding these 8 participants from the total BT sample showed a similarly significant reduction on the YGTSS-TTSS as in the main model (coefficient [95% CI] − 7.60 [− 9.34 to − 5.85], *p *< 0.001, bootstrapped *d *= 1.01 [0.76 to 1.26]).

Mixed-effects regression analyses for the YGTSS Impairment Score and the CGAS both showed significant improvements at post-treatment for the total BT sample and each BT modality separately (Table 2 and Supplementary Tables S5–S8).

### Self- and Parent-Reported Measures

Mixed-effects regression analyses for the PTQ, the GTS-QOL, the OCI-CV, the CDI-S and the SMFQ-CV combined to *z*-scores, the SMFQ-PV, the WSAS-Y, and the WSAS-P, showed significant improvements at post-treatment for the total BT sample (Table 2 and Supplementary Table S5). The results for the specific BT modalities are presented in Supplementary Tables S2–S4 and S6–S8, but should be interpreted with caution given the small sample sizes.

### Long-Term Follow-Up

**Fig. 2:**
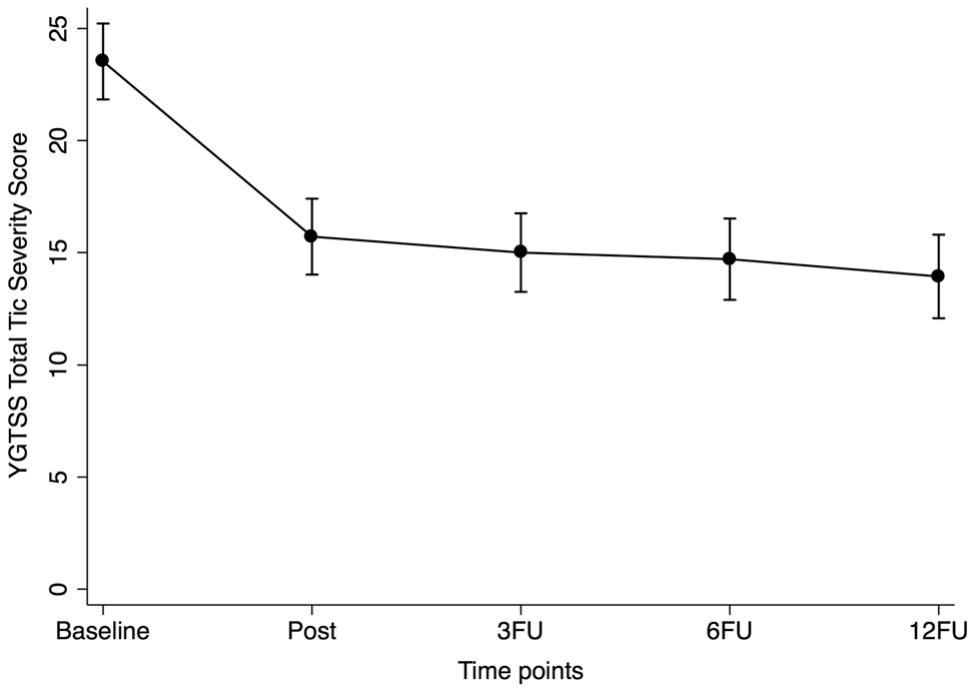
Estimated means on the YGTSS-TTSS for the total BT sample, from a mixed-effects regression model including all five time points. Error bars indicate 95% confidence intervals. *BT* behaviour therapy, *YGTSS* Yale Global Tic Severity Scale, *YGTSS-TTSS* Yale Global Tic Severity Scale-Total Tic Severity Score

Mixed-effects regression analyses showed a significant improvement between post-treatment and the 12-month follow-up on the YGTSS-TTSS for the total BT sample (coefficient [95% CI] − 1.90 [− 3.60 to − 0.20], *p *= 0.029). Similarly, there was a continued improvement on the YGTSS Impairment Score and the CGAS for the total BT sample (Table 3 and Supplementary Table S9). A third model including all five time points was also implemented in order to obtain a graphical representation of the YGTSS-TTSS for the total BT sample from baseline to the 12-month follow-up (coefficient [95% CI] − 9.60 [− 11.35 to − 7.84], *p *< 0.001; Fig. 2).

**Table 3:** Detailed statistics for TS/CTD-specific measures for the long-term follow-up period for the total BT sample

	Total BT (*N *= 74)		
	Mean (SE)^A^	Within-group difference^B^	Within-group effect size^C^
		Coefficient (95% CI); *p*-value	Bootstrapped *d* (95% CI)
Yale global tic severity scale			
Total tic severity score			
Post-treatment (*n *= 71)	15.72 (0.88)		
3-month follow-up (*n *= 63)	14.97 (0.91)	− 0.75 (− 2.33 to 0.84); *p *= 0.356	0.12 (− 0.08 to 0.31)
6-month follow-up (*n *= 56)	14.56 (0.94)	− 1.16 (− 2.80 to 0.49); *p *= 0.169	0.16 (− 0.04 to 0.36)
12-month follow-up (n = 51)	13.82 (0.97)	− 1.90 (− 3.60 to − 0.20); *p *= 0.029*	0.24 (0.03 to 0.45)
Motor tic severity score			
Post-treatment (*n *= 71)	9.57 (0.50)		
3-month follow-up (*n *= 63)	8.87 (0.52)	− 0.70 (− 1.68 to 0.27); *p *= 0.159	0.17 (− 0.04 to 0.38)
6-month follow-up (*n *= 56)	8.91 (0.54)	− 0.66 (− 1.68 to 0.35); *p *= 0.200	0.16 (− 0.09 to 0.40)
12-month follow-up (n = 51)	8.50 (0.56)	− 1.08 (− 2.12 to − 0.03); *p *= 0.044*	0.23 (− 0.08 to 0.53)
Vocal tic severity score			
Post-treatment (*n *= 71)	6.15 (0.55)		
3-month follow-up (*n *= 63)	6.10 (0.57)	− 0.05 (− 1.02 to 0.92); *p *= 0.920	0.04 (− 0.17 to 0.25)
6-month follow-up (*n *= 56)	5.65 (0.58)	− 0.50 (− 1.51 to 0.51); *p *= 0.332	0.11 (− 0.11 to 0.34)
12-month follow-up (n = 51)	5.30 (0.60)	− 0.85 (− 1.89 to 0.20); *p *= 0.111	0.19 (− 0.03 to 0.41)
Impairment score			
Post-treatment (*n *= 71)	9.33 (1.02)		
3-month follow-up (*n *= 63)	7.16 (1.07)	− 2.17 (− 4.21 to − 0.13); *p *= 0.037*	0.23 (0.02 to 0.44)
6-month follow-up (*n *= 56)	7.54 (1.11)	− 1.79 (− 3.91 to 0.33); *p *= 0.098	0.20 (− 0.02 to 0.42)
12-month follow-up (n = 51)	6.75 (1.14)	− 2.57 (− 4.76 to − 0.38); *p *= 0.021*	0.27 (0.03 to 0.48)
Parent tic questionnaire			
Post-treatment (*n *= 53)	22.42 (2.25)		
3-month follow-up (*n *= 45)	21.01 (2.40)	− 1.41 (− 6.46 to 3.63); *p *= 0.583	0.12 (− 0.18 to 0.41)
6-month follow-up (*n *= 40)	22.02 (2.51)	− 0.41 (− 5.64 to 4.83); *p *= 0.879	0.05 (− 0.28 to 0.38)
12-month follow-up (n = 35)	19.25 (2.64)	− 3.17 (− 8.66 to 2.31); *p *= 0.257	0.15 (− 0.17 to 0.46)
GTS-quality of life scale			
Post-treatment (*n *= 40)	15.01 (2.03)		
3-month follow-up (*n *= 41)	16.30 (2.02)	1.29 (− 2.51 to 5.09); *p *= 0.506	− 0.12 (− 0.35 to 0.11)
6-month follow-up (*n *= 33)	17.08 (2.15)	2.07 (− 2.02 to 6.16); *p *= 0.321	− 0.16 (− 0.43 to 0.12)
12-month follow-up (n = 28)	19.37 (2.27)	4.36 (0.01 to 8.70); *p *= 0.049*	− 0.30 (− 0.60 to 0.00)
Premonitory urge for tics scale			
Post-treatment (*n *= 46)	20.28 (0.85)		
3-month follow-up (*n *= 45)	20.38 (0.86)	0.10 (− 1.42 to 1.62); *p *= 0.893	− 0.06 (-0.31 to 0.18)
6-month follow-up (*n *= 35)	19.16 (0.92)	− 1.11 (− 2.76 to 0.53); *p *= 0.185	0.15 (− 0.09 to 0.39)
12-month follow-up (n = 29)	20.52 (0.97)	0.24 (− 1.48 to 1.96); *p *= 0.784	0.04 (− 0.25 to 0.33)

*BT* behaviour therapy,* CI* confidence interval,* CTD* chronic motor or vocal tic disorder, *GTS* Gilles de la Tourette,* SE* standard error,* TS* Tourette syndrome

^A^  Estimated means from the mixed-effects regression model

^B^  Coefficients at the 3-month, 6-month, and 12-month follow-ups compare with the post-treatment time point

^C^  Bootstrapped effect sizes (*d*) are derived from the mixed-effects regression model. Effect sizes of 0.2, 0.5, and 0.8 are considered small, moderate, and large, respectively

*Significant at an alpha level of 0.05

At the time of the data freeze, 11 participants were still active at the clinic and had not yet reached the 12-month follow-up. For the remaining 63 participants, 39 (75%, available data for *n *= 52) were classified as treatment responders at the 12-month follow-up. Numbers were too small to report on the specific BT modalities. Between the post-treatment and the 12-month follow-up time points, the 63 participants received on average 3.02 additional follow-up sessions (SD = 1.64, range = 0–10, complete data). The follow-up sessions primarily consisted of a YGTSS administration and brief clinical advice to promote the maintenance of the treatment effects. For participants who received more than 3 follow-up sessions, the additional sessions were considered as pure booster sessions focused on improving TS/CTD symptoms.

Additionally, comparing data from the post-treatment and the 12-month follow-up time points, 4 out of 74 participants (5%; available data for *n *= 53) changed their medication (α2-agonists or antipsychotics): 2 participants (3%) either increased their dosage or started medication, 1 participant (1%) switched from one compound to another, and 1 participant (1%) decreased his dosage. A mixed-effects regression analysis excluding these 4 participants from the total BT sample showed a similar continued improvement between the post-treatment and the 12-month follow-up time points on the YGTSS-TTSS as in the main model (coefficient [95% CI]  − 1.86 [− 3.64 to − 0.07], *p *= 0.041).

Regarding the self- and parent-reported measures, mixed-effects regression analyses showed that the gains were maintained (i.e., unchanged) between post-treatment and the 12-month follow-up on the PTQ, the PUTS, the OCI-CV, the SMFQ-PV, the WSAS-Y, and the WSAS-P. For the GTS-QOL and the CDI-S and the SMFQ-CV combined to *z*-scores, mixed-effects regression analyses showed that symptoms significantly deteriorated between the same time points. Full details are presented in Table 3 and Supplementary Table S9. Note that long-term follow-up analyses only were performed for the total BT sample due to an increased amount of missing data in the later time points.

## Discussion

This study evaluated the effectiveness of BT for young people with TS/CTD in a naturalistic setting, a TS/CTD specialist outpatient clinic in Stockholm, Sweden. Additionally, the study examined the long-term maintenance (up to 1 year after the end of treatment) of the treatment effects, regardless of whether the participants had initially responded to treatment.

Baseline participant characteristics were in many respects consistent with existing literature [9, 11, 12, 37], including the age of tic onset, mean age, sex ratio, type of tic disorder ratio, and the mean baseline YGTSS-TTSS score. A majority of the participants received ERP (62%) compared to HRT (19%) and *other BT* (19%). The therapists’ preference for ERP may have been influenced by various factors, including their participation in an ERP training workshop during the study period, their familiarity with ERP from treating other related disorders (primarily obsessive–compulsive disorder), and that in Europe ERP is generally more frequently used than HRT/CBIT.

Tic severity and tic-related impairment (as measured by the YGTSS) improved significantly for both the total BT sample and each separate BT modality, with within-group effect sizes consistently in the large range. The estimated reduction of 7.7 raw points on the YGTSS-TTSS for the total BT sample at post-treatment was in line with several major RCTs of BT for TS/CTD (range = 3.4-8.5 raw points) [9, 11, 12]. Further, the 7.7 raw points equals an average tic severity reduction of 33%, which is well above the proposed 25% reduction corresponding to a clinically meaningful change [38]. The treatment response rate of 57% at post-treatment was comparable to the 53% reported for CBIT/HRT in the largest RCT to date [9]. Thus, we did not observe worse outcomes in a naturalistic setting, compared to controlled settings, as reported for other common psychiatric disorders [15, 16].

Results from self- and parent-reported measures were also generally positive, with significant improvements on measures of tic severity, TS/CTD-specific health-related quality of life, obsessive–compulsive symptoms, depression, and global functional impairment. More specifically, the estimated reduction of 14.6 raw points on the PTQ was above the proposed 10-point reduction indicative for positive clinical change [39]. Percentage wise, however, the 39% reduction on the PTQ was below the proposed 45% to 55% range for positive treatment response [39]. Similarly to previous trials of BT for TS/CTD [18, 40, 41], we did not observe significant improvements on the PUTS, suggesting that premonitory urges are not amenable to modification with BT.

Regarding long-term durability of the treatment effects, tic severity and tic-related impairment (as measured by the YGTSS) improved further between post-treatment and the 12-month follow-up. The treatment response rate for the total BT sample increased from 57% at post-treatment to 75% at the 12-month follow-up. Taken together, these results indicate that the effects of BT delivered in a naturalistic specialist clinical setting are maintained for at least 1 year after the end of treatment. Thus, this study provides important new information, as published RCTs have typically not followed patients for longer than 6 months after the end of treatment and have only followed treatment responders [9, 11].

This study had several strengths, primarily related to its naturalistic design and the inclusion of a long-term follow-up. External validity was arguably higher than in the typical RCT given that treatment was provided in a naturalistic setting, no specific eligibility criteria were used (e.g., 11% of the sample had comorbid autism spectrum disorder), and concurrent interventions (such as medication) were allowed. However, this study also had limitations. By definition, the naturalistic design did not control for the natural passage of time, meaning that participants could potentially have improved due to reasons unrelated to the interventions provided, such as ‘regression to the mean’ or the natural waxing and waning of tics. The study is further limited by missing data, especially for self- and parent-reported measures and later follow-up time points. Clinician-rated measures (including the YGTSS) were administered by the therapists providing treatment, rather than independent assessors. A small proportion of participants were on TS/CTD medication or changed their medication during the study period, but sensitivity analyses excluding these participants did not modify the results. We recruited a consecutive sample of patients referred to our clinic but the proportion of individuals who did not consent to participation is unknown because we did not have ethical approval to save their data. However, in our experience, the vast majority of families agreed to be included in the study. Lastly, it is important to note that, even if the study design was naturalistic, the patients were still seen in a specialist setting and treated by trained therapists using evidence-based treatment manuals. The results might therefore not be generalisable to general CAMHS or other healthcare settings.

## Summary

To our knowledge, the current study is the largest to date evaluating BT for young people with TS/CTD in a naturalistic setting. The results confirmed that BT is an effective and durable treatment for young people with TS/CTD in a specialist clinical context, with effects comparable to those reported in RCTs.

## Electronic supplementary material

Below is the link to the electronic supplementary material.

Supplementary material 1 (PDF 856 kb)

## Data Availability

The data are pseudonymised according to national (Swedish) and EU legislation, and cannot be anonymised and published in an open repository. The data can be made available upon reasonable request on a case by case basis according to the current legislation and ethical permits.
