# Supplementary material for: Effectiveness of Behaviour Therapy for Children and Adolescents with Tourette Syndrome and Chronic Tic Disorder in a Naturalistic Setting
Source: Child Psychiatry Hum Dev. 2020 Dec 14;52(4):739–50. doi: 10.1007/s10578-020-01098-y (PMC8238753; doi:10.1007/s10578-020-01098-y)
Supplement: Supplementary file 1 — Supplementary material 1 (PDF 856 kb) [file 10578_2020_1098_MOESM1_ESM.pdf]

Effectiveness of behaviour therapy for children and adolescents with Tourette syndrome and chronic tic disorder in a naturalistic setting

Per Andrén, *MSc*,[1,2]* Vera Wachtmeister, *MSc*,[2] Julia Franzé, *MSc*,[3] Caroline Speiner, *MSc*,[3] Lorena Fernández de la Cruz, *PhD*,[1,2] Erik Andersson, *PhD*,[1,2,3] Elles de Schipper, *PhD*,[1,2] Daniel Rautio, *MSc*,[1,2] Maria Silverberg-Mörse, *MD*,[2] Eva Serlachius, *MD, PhD*,[1,2] and David Mataix-Cols, *PhD*[1,2]

[1]Centre for Psychiatry Research, Department of Clinical Neuroscience, Karolinska Institutet, Stockholm, Sweden

[2]Stockholm Health Care Services, Region Stockholm, Stockholm, Sweden

[3]Department of Clinical Neuroscience, Division of Psychology, Karolinska Institutet, Stockholm, Sweden

***Correspondence to:** Per Andrén, Karolinska Institutet, Department of Clinical Neuroscience; Child and Adolescent Psychiatry Research Center, Gävlegatan 22, 113 30 Stockholm, Sweden. E-mail: per.andren@ki.se.

Supplementary Table 1. The YGTSS checklist of current (within the last week) motor and vocal tics at baseline for the total BT sample and by BT modality.

	**Total BT (*N*=74)**	**ERP (*n*=46)**	**HRT (*n*=14)**	**Other BT (*n*=14)**
**Motor tics**, *n* (%) (*n*=71)				
Any motor tic	70 (99)	43 (98)	14 (100)	13 (100)
Eye movements	53 (75)	33 (75)	11 (79)	9 (69)
Nose, mouth, tongue movements, or facial grimacing	45 (63)	27 (61)	7 (50)	11 (85)
Head jerks/movements	47 (66)	32 (73)	7 (50)	8 (62)
Shoulder jerks/movements	29 (41)	19 (43)	7 (50)	3 (23)
Arm or hand movements	48 (68)	29 (66)	8 (57)	11 (85)
Leg, foot or toe movements	40 (56)	27 (61)	8 (57)	5 (38)
Abdominal/trunk/pelvis movements	19 (27)	14 (32)	3 (21)	2 (15)
Rude/obscene gestures; obscene finger/hand gestures	4 (6)	1 (2)	0 (0)	3 (23)
Copying the action of another	0 (0)	0 (0)	0 (0)	0 (0)
Sudden tic-like impulsive behaviours	2 (3)	2 (5)	0 (0)	0 (0)
Tic-like behaviours that could injure/mutilate others	1 (1)	1 (2)	0 (0)	0 (0)
Self-injurious tic-like behaviour(s)	3 (4)	3 (7)	0 (0)	0 (0)
Other simple motor tics	15 (21)	12 (27)	1 (7)	2 (15)
Other complex motor tics	18 (25)	13 (30)	3 (21)	2 (15)
**Vocal tics**, *n* (%) (*n*=71)				
Any vocal tic	56 (79)	36 (82)	10 (71)	10 (77)
Coughing	17 (24)	10 (23)	4 (29)	3 (23)
Throat clearing	36 (51)	26 (59)	8 (57)	2 (15)
Sniffing	27 (38)	20 (45)	2 (14)	5 (38)
Whistling	5 (7)	3 (7)	2 (14)	0 (0)
Animal or bird noises	6 (8)	5 (11)	0 (0)	1 (8)
Syllables	8 (11)	5 (11)	0 (0)	3 (23)
Words	4 (6)	1 (2)	1 (7)	2 (15)
Rude or obscene words or phrases	3 (4)	2 (5)	0 (0)	1 (8)
Repeating what someone else said	6 (8)	3 (7)	1 (7)	2 (15)
Repeating something the patient said	5 (7)	4 (9)	1 (7)	0 (0)
Other tic-like speech problems	1 (1)	0 (0)	0 (0)	1 (8)
Other simple vocal tics	31 (44)	23 (52)	2 (14)	6 (46)
Other complex vocal tics	3 (4)	2 (5)	0 (0)	1 (8)

Abbreviations: BT = behaviour therapy; ERP = exposure with response prevention; HRT = habit reversal training; YGTSS = Yale Global Tic Severity Scale.

Supplementary Table 2. Detailed statistics for TS/CTD-specific measures at post-treatment for the ERP modality.

	ERP (*n*=46)		
	**Mean (SE)[A]**	**Within-group difference Coefficient (95% CI); *p*-value**	**Within-group effect size[B] Bootstrapped *d* (95% CI)**
**Yale Global Tic Severity Scale**			
Total Tic Severity Score			
Baseline (*n*=44)	23.76 (1.09)		
Post-treatment (*n*= 44)	15.86 (1.09)	-7.90 (-9.81 to -5.99); *p*<0.001*	1.09 (0.75 to 1.43)
Motor Tic Severity Score			
Baseline (*n*= 44)	14.08 (0.61)		
Post-treatment (*n*= 44)	9.46 (0.61)	-4.62 (-5.82 to -3.43); *p*<0.001*	1.11 (0.68 to 1.54)
Vocal Tic Severity Score			
Baseline (*n*= 44)	9.67 (0.75)		
Post-treatment (*n*= 44)	6.40 (0.75)	-3.28 (-4.46 to -2.10); *p*<0.001*	0.66 (0.36 to 0.97)
Impairment Score			
Baseline (*n*= 44)	23.30 (1.34)		
Post-treatment (*n*= 44)	9.51 (1.34)	-13.76 (-16.56 to 10.96); *p*<0.001*	1.59 (1.16 to 2.02)
**Parent Tic Questionnaire**			
Baseline (*n*= 44)	37.67 (2.80)		
Post-treatment (*n*= 35)	26.39 (3.02)	-11.27 (-16.91 to -5.62); *p*<0.001*	0.57 (0.23 to 0.91)
**GTS - Quality of Life Scale**			
Baseline (*n*= 36)	30.01 (2.78)		
Post-treatment (*n*= 29)	13.51 (3.02)	-16.43 (-22.58 to -10.28); *p*<0.001*	1.03 (0.60 to 1.46)
**Premonitory Urge for Tics Scale**			
Baseline (*n*= 42)	19.81 (0.94)		
Post-treatment (*n*= 31)	19.60 (1.06)	-0.21 (-2.58 to 2.17); *p*=0.865	0.03 (-0.40 to 0.46)

Note: * = Significant at an alpha level of 0.05; A = Estimated means from the mixed-effects regression model; B = Bootstrapped effect sizes (*d*) are derived from the mixed-effects regression model. Effect sizes of 0.2, 0.5, and 0.8 are considered small, moderate, and large, respectively.

Abbreviations: CI = confidence interval; CTD = chronic motor or vocal tic disorder; ERP = exposure with response prevention; GTS = Gilles de la Tourette; SE = standard error; TS = Tourette syndrome.

Supplementary Table 3. Detailed statistics for TS/CTD-specific measures at post-treatment for the HRT modality.

	HRT (*n*=14)		
	**Mean (SE)[A]**	**Within-group difference Coefficient (95% CI); *p*-value**	**Within-group effect size[B] Bootstrapped *d* (95% CI)**
**Yale Global Tic Severity Scale**			
Total Tic Severity Score			
Baseline (*n*= 14)	23.36 (1.95)		
Post-treatment (*n*= 13)	17.24 (2.00)	-6.09 (-9.87 to -2.31); *p*=0.002*	0.82 (0.19 to 1.44)
Motor Tic Severity Score			
Baseline (*n*= 14)	14.93 (1.08)		
Post-treatment (*n*= 13)	11.16 (1.11)	-3.77 (-5.89 to -1.65); *p*<0.001*	0.99 (0.20 to 1.77)
Vocal Tic Severity Score			
Baseline (*n*= 14)	8.43 (1.33)		
Post-treatment (*n*= 13)	6.05 (1.36)	-2.33 (-4.81 to 0.15); *p*=0.065	0.48 (-0.14 to 1.11)
Impairment Score			
Baseline (*n*= 14)	22.14 (2.37)		
Post-treatment (*n*= 13)	10.69 (2.44)	-12.07 (-15.22 to -8.92); *p*<0.001*	1.19 (0.00 to 2.37)
**Parent Tic Questionnaire**			
Baseline (*n*= 13)	37.54 (5.19)		
Post-treatment (*n*= 12)	16.16 (5.33)	-21.25 (-29.64 to -12.86); *p*<0.001*	1.37 (0.62 to 2.13)
**GTS - Quality of Life Scale**			
Baseline (*n*= 9)	30.11 (5.61)		
Post-treatment (*n*= 7)	21.76 (6.17)	-8.42 (-17.33 to 0.49); *p*=0.064	0.49 (0.06 to 0.91)
**Premonitory Urge for Tics Scale**			
Baseline (*n*= 12)	20.17 (1.76)		
Post-treatment (*n*= 10)	21.49 (1.89)	1.34 (-0.90 to 3.57); *p*=0.241	-0.35 (-1.06 to 0.36)

Note: * = Significant at an alpha level of 0.05; A = Estimated means from the mixed-effects regression model; B = Bootstrapped effect sizes (*d*) are derived from the mixed-effects regression model. Effect sizes of 0.2, 0.5, and 0.8 are considered small, moderate, and large, respectively.

Abbreviations: CI = confidence interval; CTD = chronic motor or vocal tic disorder; GTS = Gilles de la Tourette; HRT = habit reversal training; SE = standard error; TS = Tourette syndrome.

Supplementary Table 4. Detailed statistics for TS/CTD-specific measures at post-treatment for the *other BT* modality.

	Other BT (*n*=14)		
	**Mean (SE)[A]**	**Within-group difference Coefficient (95% CI); *p*-value**	**Within-group effect size[B] Bootstrapped *d* (95% CI)**
**Yale Global Tic Severity Scale**			
Total Tic Severity Score			
Baseline (*n*= 13)	22.45 (2.00)		
Post-treatment (*n*= 14)	13.64 (1.95)	-8.81 (-12.72 to -4.91); *p*<0.001*	1.13 (0.59 to 1.66)
Motor Tic Severity Score			
Baseline (*n*= 13)	14.17 (1.11)		
Post-treatment (*n*= 14)	8.36 (1.08)	-5.95 (-8.71 to -3.19); *p*<0.001*	1.50 (0.75 to 2.25)
Vocal Tic Severity Score			
Baseline (*n*= 13)	8.35 (1.36)		
Post-treatment (*n*= 14)	5.29 (1.33)	-3.07 (-5.66 to -0.49); *p*=0.020*	0.54 (0.08 to 1.01)
Impairment Score			
Baseline (*n*= 13)	16.65 (2.44)		
Post-treatment (*n*= 14)	7.14 (2.37)	-9.63 (-14.54 to -4.71); *p*<0.001*	1.23 (0.52 to 1.95)
**Parent Tic Questionnaire**			
Baseline (*n*= 12)	37.44 (5.33)		
Post-treatment (*n*= 6)	16.82 (6.91)	-20.86 (-35.32 to -6.41); *p*=0.005*	0.91 (0.01 to 1.82)
**GTS - Quality of Life Scale**			
Baseline (*n*= 12)	35.72 (4.81)		
Post-treatment (*n*= 4)	26.28 (7.72)	-3.68 (-18.24 to 10.89); *p*=0.621	0.26 (-0.37 to 0.89)
**Premonitory Urge for Tics Scale**			
Baseline (*n*= 13)	20.21 (1.68)		
Post-treatment (*n*= 5)	19.57 (2.53)	1.18 (-1.23 to 3.59); *p*=0.337	-0.15 (-0.51 to 0.21)

Note: * = Significant at an alpha level of 0.05; A = Estimated means from the mixed-effects regression model; B = Bootstrapped effect sizes (*d*) are derived from the mixed-effects regression model. Effect sizes of 0.2, 0.5, and 0.8 are considered small, moderate, and large, respectively.

Abbreviations: BT = behaviour therapy; CI = confidence interval; CTD = chronic motor or vocal tic disorder; GTS = Gilles de la Tourette; SE = standard error; TS = Tourette syndrome.

Supplementary Table 5. Detailed statistics for non-TS/CTD-specific measures at post-treatment for the total BT sample.

	Total BT (*N*=74)		
	**Mean (SE)[A]**	**Within-group difference Coefficient (95% CI); *p*-value**	**Within-group effect size[B] Bootstrapped *d* (95% CI)**
**CGAS**			
Baseline (*n*=74)	56.31 (1.01)		
Post-treatment (*n*=71)	63.26 (1.02)	6.95 (5.25 to 8.65); *p*<0.001*	0.80 (0.62 to 0.97)
**OCI-CV**			
Baseline (*n*=66)	8.64 (0.69)		
Post-treatment (*n*=44)	6.97 (0.76)	-1.67 (-2.77 to -0.58); *p*=0.003*	0.29 (0.08 to 0.50)
**CDI-S/SMFQ-CV, comb. *z*-score**			
Baseline (*n*=60)	0.24 (0.13)		
Post-treatment (*n*=43)	-0.19 (0.14)	-0.42 (-0.71 to -0.14); *p*=0.003*	0.42 (0.13 to 0.71)
**SMFQ-PV**			
Baseline (*n*=66)	6.98 (0.59)		
Post-treatment (*n*=51)	4.46 (0.66)	-2.52 (-3.94 to -1.11); *p*<0.001*	0.52 (0.22 to 0.83)
**WSAS-Y**			
Baseline (*n*=64)	9.70 (0.91)		
Post-treatment (*n*=46)	5.74 (1.06)	-3.96 (-6.39 to -1.53); *p*=0.001*	0.51 (0.18 to 0.85)
**WSAS-P**			
Baseline (*n*=70)	12.78 (0.91)		
Post-treatment (*n*=54)	7.61 (1.02)	-5.17 (-7.26 to -3.07); *p*<0.001*	0.65 (0.37 to 0.92)

Note: * = Significant at an alpha level of 0.05; A = Estimated means from the mixed-effects regression model; B = Bootstrapped effect sizes (*d*) are derived from the mixed-effects regression model. Effect sizes of 0.2, 0.5, and 0.8 are considered small, moderate, and large, respectively.

Abbreviations: BT = behaviour therapy; CDI-S = Children's Depression Inventory - Short version; CGAS = Children's Global Assessment Scale; CI = confidence interval; comb. = combined; CTD = chronic motor or vocal tic disorder; OCI-CV = Obsessive Compulsive Inventory - Child Version; SE = standard error; SMFQ-CV = Short Mood and Feelings Questionnaire – Child Version; SMFQ-PV = Short Mood and Feelings Questionnaire – Parent Version; TS = Tourette syndrome; WSAS-P = Work and Social Adjustment Scale – Parent version; WSAS-Y = Work and Social Adjustment Scale – Youth version.

Supplementary Table 6. Detailed statistics for non-TS/CTD-specific measures at post-treatment for the ERP modality.

	ERP (*n*=46)		
	**Mean (SE)[A]**	**Within-group difference Coefficient (95% CI); *p*-value**	**Within-group effect size[B] Bootstrapped *d* (95% CI)**
**CGAS**			
Baseline (*n*=46)	55.43 (1.26)		
Post-treatment (*n*= 44)	64.59 (1.27)	9.17 (6.87 to 11.47); *p*<0.001*	1.15 (0.89 to 1.41)
**OCI-CV**			
Baseline (*n*=41)	8.29 (0.87)		
Post-treatment (*n*=29)	6.51 (0.94)	-1.83 (-3.23 to -0.44); *p*=0.010*	0.36 (0.09 to 0.64)
**CDI-S/SMFQ-CV, comb. *z*-score**			
Baseline (*n*=38)	0.08 (0.15)		
Post-treatment (*n*=29)	-0.41 (0.17)	-0.49 (-0.82 to -0.16); *p*=0.003*	0.57 (0.09 to 1.05)
**SMFQ-PV**			
Baseline (*n*=43)	7.37 (0.70)		
Post-treatment (*n*=32)	3.85 (0.80)	-3.53 (-5.38 to -1.68); *p*<0.001*	0.66 (0.33 to 1.00)
**WSAS-Y**			
Baseline (*n*=40)	9.80 (1.13)		
Post-treatment (*n*=31)	4.90 (1.27)	-4.90 (-7.78 to -2.03); *p*=0.001*	0.67 (0.30 to 1.03)
**WSAS-P**			
Baseline (*n*=44)	13.02 (1.12)		
Post-treatment (*n*=34)	7.22 (1.25)	-5.78 (-8.34 to -3.23); *p*<0.001*	0.73 (0.44 to 1.03)

Note: * = Significant at an alpha level of 0.05; A = Estimated means from the mixed-effects regression model; B = Bootstrapped effect sizes (*d*) are derived from the mixed-effects regression model. Effect sizes of 0.2, 0.5, and 0.8 are considered small, moderate, and large, respectively.

Abbreviations: CDI-S = Children's Depression Inventory - Short version; CGAS = Children's Global Assessment Scale; CI = confidence interval; comb. = combined; CTD = chronic motor or vocal tic disorder; ERP = exposure with response prevention; OCI-CV = Obsessive Compulsive Inventory - Child Version; SE = standard error; SMFQ-CV = Short Mood and Feelings Questionnaire – Child Version; SMFQ-PV = Short Mood and Feelings Questionnaire – Parent Version; TS = Tourette syndrome; WSAS-P = Work and Social Adjustment Scale – Parent version; WSAS-Y = Work and Social Adjustment Scale – Youth version.

Supplementary Table 7. Detailed statistics for non-TS/CTD-specific measures at post-treatment for the HRT modality.

	HRT (*n*=14)		
	**Mean (SE)[A]**	**Within-group difference Coefficient (95% CI); *p*-value**	**Within-group effect size[B] Bootstrapped *d* (95% CI)**
**CGAS**			
Baseline (*n*=14)	57.71 (2.28)		
Post-treatment (*n*=13)	61.13 (2.32)	3.58 (1.84 to 5.31); *p*<0.001*	0.38 (0.10 to 0.66)
**OCI-CV**			
Baseline (*n*=12)	7.92 (1.61)		
Post-treatment (*n*=10)	6.28 (1.67)	-1.61 (-3.96 to 0.74); *p*=0.179	0.31 (-0.26 to 0.89)
**CDI-S/SMFQ-CV, comb. *z*-score**			
Baseline (*n*=10)	0.17 (0.29)		
Post-treatment (*n*=10)	0.13 (0.29)	-0.02 (-0.56 to 0.53); *p*=0.950	0.02 (-0.58 to 0.61)
**SMFQ-PV**			
Baseline (*n*=11)	4.36 (1.37)		
Post-treatment (*n*=12)	3.19 (1.32)	-1.07 (-2.12 to -0.03); *p*=0.045*	0.40 (0.03 to 0.84)
**WSAS-Y**			
Baseline (*n*=11)	8.34 (2.15)		
Post-treatment (*n*=10)	5.71 (2.25)	-2.94 (-8.29 to 2.42); *p*=0.283	0.35 (-0.33 to 1.02)
**WSAS-P**			
Baseline (*n*=13)	9.23 (2.07)		
Post-treatment (*n*=12)	5.67 (2.14)	-3.51 (-7.88 to 0.85); *p*=0.115	0.55 (-0.10 to 1.20)

Note: * = Significant at an alpha level of 0.05; A = Estimated means from the mixed-effects regression model; B = Bootstrapped effect sizes (*d*) are derived from the mixed-effects regression model. Effect sizes of 0.2, 0.5, and 0.8 are considered small, moderate, and large, respectively.

Abbreviations: CDI-S = Children's Depression Inventory - Short version; CGAS = Children's Global Assessment Scale; CI = confidence interval; comb. = combined; CTD = chronic motor or vocal tic disorder; HRT = habit reversal training; OCI-CV = Obsessive Compulsive Inventory - Child Version; SE = standard error; SMFQ-CV = Short Mood and Feelings Questionnaire – Child Version; SMFQ-PV = Short Mood and Feelings Questionnaire – Parent Version; TS = Tourette syndrome; WSAS-P = Work and Social Adjustment Scale – Parent version; WSAS-Y = Work and Social Adjustment Scale – Youth version.

Supplementary Table 8. Detailed statistics for non-TS/CTD-specific measures at post-treatment for the *other BT* modality.

	Other BT (*n*=14)		
	**Mean (SE)[A]**	**Within-group difference Coefficient (95% CI); *p*-value**	**Within-group effect size[B] Bootstrapped *d* (95% CI)**
**CGAS**			
Baseline (*n*=14)	57.79 (2.28)		
Post-treatment (*n*=14)	61.07 (2.28)	3.29 (0.35 to 6.23); *p*=0.028*	0.32 (0.01 to 0.63)
**OCI-CV**			
Baseline (*n*=13)	10.26 (1.51)		
Post-treatment (*n*=5)	9.66 (1.99)	-0.28 (-1.54 to 0.99); *p*=0.669	0.03 (-0.09 to 0.16)
**CDI-S/SMFQ-CV, comb. *z*-score**			
Baseline (*n*=12)	0.74 (0.27)		
Post-treatment (*n*=4)	0.25 (0.43)	-0.45 (-1.41 to 0.52); *p*=0.362	0.34 (-0.33 to 1.00)
**SMFQ-PV**			
Baseline (*n*=12)	7.89 (1.32)		
Post-treatment (*n*=7)	9.04 (1.67)	0.90 (-2.62 to 4.42); *p*=0.617	-0.15 (-0.89 to 0.58)
**WSAS-Y**			
Baseline (*n*=13)	10.44 (1.98)		
Post-treatment (*n*=5)	10.87 (3.13)	0.54 (-6.91 to 8.00); *p*=0.886	-0.05 (-1.13 to 1.03)
**WSAS-P**			
Baseline (*n*=13)	15.39 (2.06)		
Post-treatment (*n*=8)	11.33 (2.54)	-3.56 (-8.83 to 1.70); *p*=0.185	0.38 (-0.33 to 1.08)

Note: * = Significant at an alpha level of 0.05; A = Estimated means from the mixed-effects regression model; B = Bootstrapped effect sizes (*d*) are derived from the mixed-effects regression model. Effect sizes of 0.2, 0.5, and 0.8 are considered small, moderate, and large, respectively.

Abbreviations: BT = behaviour therapy; CDI-S = Children's Depression Inventory - Short version; CGAS = Children’s Global Assessment Scale; CI = confidence interval; comb. = combined; CTD = chronic motor or vocal tic disorder; OCI-CV = Obsessive Compulsive Inventory - Child Version; SE = standard error; SMFQ-CV = Short Mood and Feelings Questionnaire – Child Version; SMFQ-PV = Short Mood and Feelings Questionnaire – Parent Version; TS = Tourette syndrome; WSAS-P = Work and Social Adjustment Scale – Parent version; WSAS-Y = Work and Social Adjustment Scale – Youth version.

Supplementary Table 9. Detailed statistics for non-TS/CTD-specific measures for the long-term follow-up period for the total BT sample.

	Total BT (*N*=74)		
	**Mean (SE)[A]**	**Within-group difference Coefficient (95% CI); *p*-value**	**Within-group effect size[B] Bootstrapped *d* (95% CI)**
**CGAS**			
Post-treatment (*n*=71)	63.19 (1.12)		
3-month follow-up (*n*=63)	63.42 (1.14)	0.22 (-1.17 to 1.61); *p*=0.755	0.02 (-0.09 to 0.14)
6-month follow-up (*n*=56)	64.07 (1.16)	0.88 (-0.57 to 2.32); *p*=0.233	0.09 (-0.05 to 0.23)
12-month follow-up (n=52)	65.53 (1.17)	2.34 (0.85 to 3.82); *p*=0.002*	0.23 (0.04 to 0.42)
**OCI-CV**			
Post-treatment (*n*=44)	6.36 (0.72)		
3-month follow-up (*n*=44)	5.81 (0.72)	-0.55 (-1.76 to 0.67); *p*=0.378	0.07 (-0.11 to 0.25)
6-month follow-up (*n*=34)	4.94 (0.76)	-1.42 (-2.74 to -0.10); *p*=0.035*	0.25 (0.07 to 0.43)
12-month follow-up (n=28)	6.08 (0.81)	-0.28 (-1.68 to 1.12); *p*=0.697	0.13 (-0.22 to 0.48)
**CDI-S/SMFQ-CV, comb. *z*-score**			
Post-treatment (*n*=43)	-0.12 (0.14)		
3-month follow-up (*n*=45)	0.01 (0.14)	0.13 (-0.09 to 0.36); *p*=0.246	-0.12 (-0.39 to 0.15)
6-month follow-up (*n*=34)	-0.02 (0.15)	0.10 (-0.15 to 0.35); *p*=0.425	-0.10 (-0.37 to 0.17)
12-month follow-up (n=28)	0.25 (0.16)	0.38 (0.11 to 0.64); *p*=0.005*	-0.32 (-0.56 to -0.07)
**SMFQ-PV**			
Post-treatment (*n*=51)	4.17 (0.54)		
3-month follow-up (*n*=46)	3.70 (0.55)	-0.47 (-1.40 to 0.46); *p*=0.320	0.11 (-0.15 to 0.37)
6-month follow-up (*n*=38)	4.00 (0.58)	-0.16 (-1.15 to 0.83); *p*=0.749	0.04 (-0.19 to 0.27)
12-month follow-up (n=34)	4.45 (0.60)	0.29 (-0.76 to 1.33); *p*=0.591	-0.06 (-0.31 to 0.18)
**WSAS-Y**			
Post-treatment (*n*=46)	5.31 (0.84)		
3-month follow-up (*n*=47)	4.74 (0.83)	-0.57 (-2.43 to 1.29); *p*=0.551	0.11 (-0.24 to 0.46)
6-month follow-up (*n*=36)	3.84 (0.93)	-1.46 (-3.48 to 0.55); *p*=0.155	0.29 (0.01 to 0.57)
12-month follow-up (n=29)	5.29 (1.01)	-0.02 (-2.18 to 2.14); *p*=0.986	0.09 (-0.32 to 0.50)
**WSAS-P**			
Post-treatment (*n*=54)	7.50 (0.88)		
3-month follow-up (*n*=49)	6.83 (0.91)	-0.67 (-2.36 to 1.02); *p*=0.438	0.10 (-0.20 to 0.40)
6-month follow-up (*n*=41)	6.61 (0.96)	-0.89 (-2.68 to 0.90); *p*=0.331	0.14 (-0.08 to 0.35)
12-month follow-up (n=35)	7.05 (1.01)	-0.45 (-2.33 to 1.43); *p*=0.637	0.08 (-0.24 to 0.41)

Note: * = Significant at an alpha level of 0.05; A = Estimated means from the mixed-effects regression model; B = Bootstrapped

effect sizes (*d*) are derived from the mixed-effects regression model. Effect sizes of 0.2, 0.5, and 0.8 are considered small, moderate, and large, respectively.

Abbreviations: BT = behaviour therapy; CDI-S = Children's Depression Inventory - Short version; CGAS = Children’s Global Assessment Scale; CI = confidence interval; comb. = combined; CTD = chronic motor or vocal tic disorder; OCI-CV = Obsessive Compulsive Inventory - Child Version; SE = standard error; SMFQ-CV = Short Mood and Feelings Questionnaire – Child Version; SMFQ-PV = Short Mood and Feelings Questionnaire – Parent Version; TS = Tourette syndrome; WSAS-P = Work and Social Adjustment Scale – Parent version; WSAS-Y = Work and Social Adjustment Scale – Youth version.
